# Photo-supercapacitors based on nanoscaled ZnO

**DOI:** 10.1038/s41598-022-15180-z

**Published:** 2022-07-07

**Authors:** Cigdem Tuc Altaf, Ozlem Coskun, Alihan Kumtepe, Arpad Mihai Rostas, Igor Iatsunskyi, Emerson Coy, Emre Erdem, Mehmet Sankir, Nurdan Demirci Sankir

**Affiliations:** 1 Department of Materials Science and Nanotechnology Engineering, TOBB University of Economics and Technology, Sogutozu Caddesi No 43 Sogutozu, 06560 Ankara, Turkey; 2 Micro and Nanotechnology Graduate Program, TOBB University of Economics and Technology, Sogutozu Caddesi No 43 Sogutozu, 06560 Ankara, Turkey; 3 National Institute for Research and Development of Isotopic and Molecular Technologies, 67-103 Donat, PO 5 Box 700, 400293 Cluj-Napoca, Romania; 4 NanoBioMedical Centre, Adam Mickiewicz University in Poznań, Wszechnicy Piastowskiej 3, 61-614 Poznań, Poland; 5 Faculty of Engineering and Natural Sciences, Sabanci University, Orhanli, 34956 Tuzla, Istanbul Turkey

**Keywords:** Energy science and technology, Materials science, Physics

## Abstract

In this study, zinc oxide (ZnO) powders in two different morphologies, nanowire (NW) and nanoflower (NF), have been synthesized by the hydrothermal method. The eligibility of the pristine ZnO nanopowders as a photo-active material has been revealed by designing P-SC devices via the facile drop-casting method on both glass and plastic substrates in large-area applications. The impact of physical properties and especially defect structures on photo-supercapacitor (P-SC) performance have been explored. Although the dark Coulombic efficiency (CE%) of both NW and NF-based P-SC were very close to each other, the CE% of NW P-SC increased 3 times, while the CE% of NF P-SC increased 1.7 times under the UV-light. This is because the charge carriers produced under light excitation, extend the discharge time, and as confirmed by electron paramagnetic resonance, photoluminescence, and transmission electron microscopy analyses, the performance of P-SCs made from NF powders was relatively low compared to those produced from NW due to the high core defects in NF powders. The energy density of 78.1 mWh kg^−1^ obtained for NF-based P-SCs is very promising, and the capacitance retention value of almost 100% for 3000 cycles showed that the P-SCs produced from these materials were entirely stable. Compared to the literature, the P-SCs we propose in this study are essential for new generation energy storage systems, thanks to their ease of design, adaptability to mass production for large-area applications, and their ability to store more energy under illumination.

Developing advanced and innovative energy conversion and storage systems is a significant part of replacing fossil fuels with clean and renewable energy sources. In this regard, solar energy is the most abundant energy source providing a practical solution for worldwide carbon-free energy demand. Solar energy offers widespread technological application zones, including photovoltaics (PV)^1–7^, photoelectrochemical water splitting (PEC)^8–14^. photoelectrochemical redox flow batteries^15–17^, and photocatalysis^18–22^. Photo-supercapacitors (P-SC) are relatively new energy conversion/storage devices and have an increasing interest in dual-use systems that simultaneously generate and store power^23–30^. Since Miyasaka's work is based on the self-charging capacitor that can directly store the electrical energy generated by solar cells; various PV-SC integrations have been reported in the literature^29,31–38^. However, most integrated systems have suffered from the complexity of integrating two or more separate parts.

On the other hand, PEC and solid-state configurated P-SC systems stand out with ease of design and low-cost fabrication to cope with high-cost solar cell materials in terms of efficient power density^39,40^. In addition, solid-state P-SC can be designed in many alternative ways. These include three-electrode systems consisting of a photo-electrode, a charge-storage electrode, and a counter electrode; or two electrodes in which the photo-electrode and the charge-storage electrode alter the working mechanism as well as the overall performance. Solid-state (photo)supercapacitors employ an ion-conducting gel electrolyte or polymer membrane and can be designed much thinner, flexible, and light-weighted, which can be applied in various applications such as wearable and portable electronics^41^.

The critical properties of electrode materials such as bandgap utilization, light-harvesting, and charge transfer optimization are required to be unraveled for widespread applications of P-SC devices. It is also critical to build a relationship between P-SC performance and defect structure-related material parameters of the electrode. Here, we aimed to utilize ZnO nanostructures with different morphologies in both rigid and flexible solid-state P-SC devices. Although ZnO has been used as electrode material in supercapacitor applications^42–47^, there are a few reports on ZnO-based P-SC. Among these limited reports, either ZnO has been used as an electron transport layer or to build heterojunction photoelectrodes^39,48–50^. However, ZnO has a great potential in P-SC as a UV-light active material having a direct wide bandgap (3.37 eV), outstanding electron mobility (115–155 cm^2^·V^−1^·s^−1^), and a considerable exciton binding energy (60 meV)^51^. Along with these featured electronic properties, it has the great advantage of being environmental compatible and ease of synthesis having various morphologies at mild conditions^52,53^. Consequently, the capability in the alteration of physical properties such as morphology, size, surface chemistry, and defect structure, have driven many applications fields to design novel devices based on ZnO.

To our best knowledge, the performance affected by the morphology, light absorption, charge generation, and defect structure of ZnO-based P-SC has not been investigated in the literature. Therefore, revealing the correlation between the electrochemical performance and the critical properties of pristine ZnO powders would benefit future P-SC applications. Since the synthesis methods and reaction parameters are known to affect the defects in the ZnO nanoparticles responsible for photoelectrochemical, electrical, and optical properties, a tremendous effort to understand and control the defects in ZnO has been exerted to date^54–57^. Here, photoluminescence (PL), transmission electron microscopy (TEM), and electron paramagnetic resonance (EPR) has been used to characterize the defects in the ZnO powders having two different morphologies, nanoflower (NF) and nanowire (NW). EPR spectroscopy has been performed under UV illumination and dark conditions to understand the characteristics of photo-induced defects in the ZnO nanostructures.

## Results and discussion

### Structural characterization, photoluminescence, and electron paramagnetic resonance spectroscopy of the ZnO powders

The hydrothermal synthesis of ZnO provides us reasonable control over the morphology, size, and crystal structure by altering reaction parameters such as temperature, pH, and concentration of the precursors^55,58–61^. In our case, ZnO NW and NF powder samples have been successfully synthesized using Zn(NO_3_)_2_·6H_2_O as a cation source, while NH_4_OH and HMTA have been used as anion sources for NW and NF, respectively. Additionally, the ZnO NW formation occurs in a basic reaction medium (pH 8.7) due to the presence of NH_4_OH. However, the pH value of the reaction solution for ZnO NF has been adjusted to 5.4 with the addition of concentrated nitric acid. Thus, changing the anionic sources and the pH of the reaction medium resulted in two different morphologies of ZnO because of the different mechanism and nucleation rates. It is known that the growth of the rod and wire-like ZnO is much faster in a basic environment as compared to an acidic medium^62^. This phenomenon can be explained by the precipitation of zinc complexes (Zn(OH)_2_ and Zn(OH)_4_^+2^) as the zinc salts react with hydroxyl (OH^−^) ions in the reaction medium^59,63^. Howbeit, the precipitation of ZnO in an acidic medium (pH 4.8–5.4) leads to the formation of sheet-like nanostructures due to the increasing H^+^ ions, which rather react with OH^−^ ions at the surface and inhibit the growth in the c-axis^59,63,64^.

**Figure 1:**
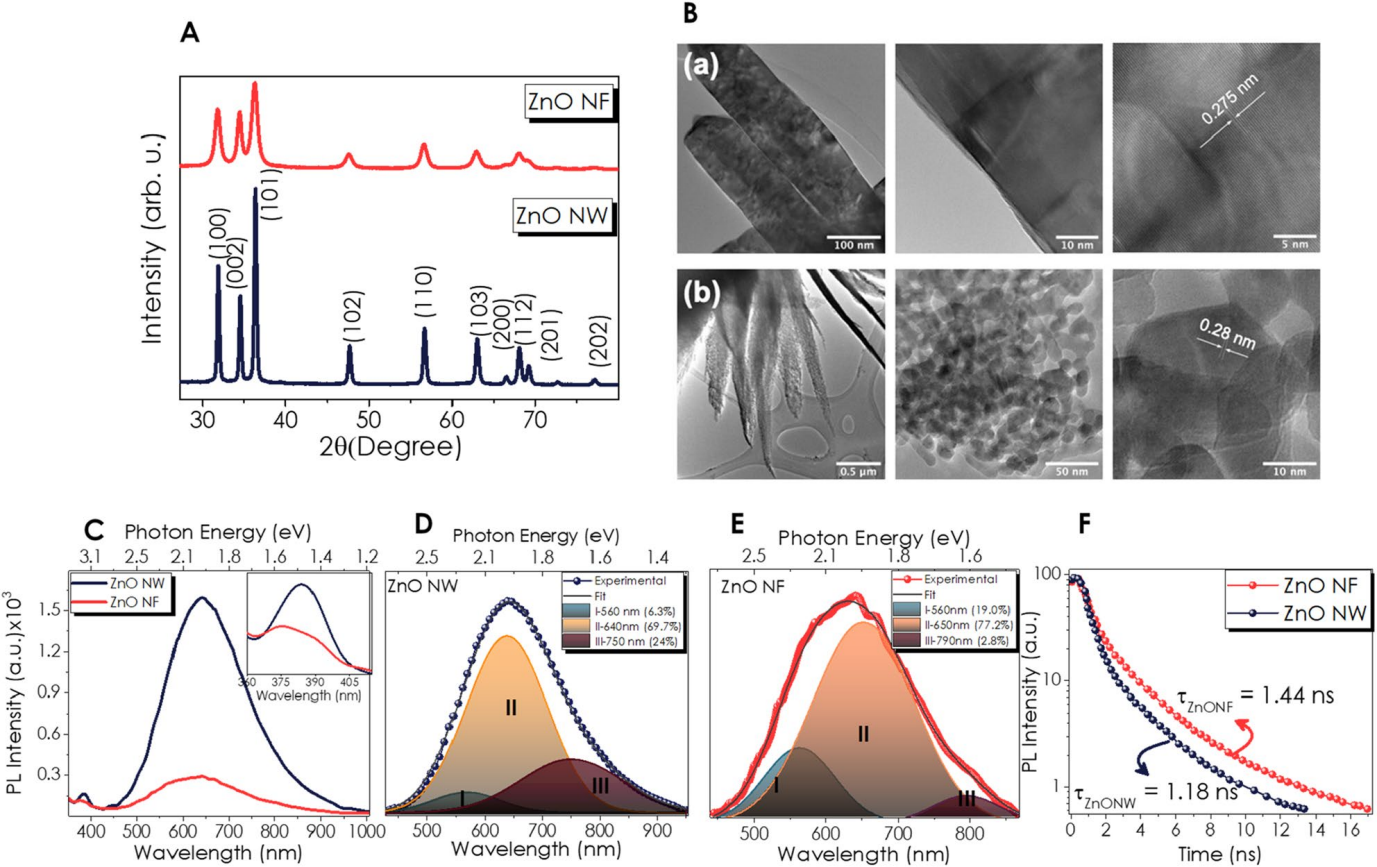
(**A**) XRD patterns; (**B**) TEM and HRTEM (**a**) NW and (**b**) NF; normalized PL spectra (**C**) Comparison of NF and NW, deconvoluted DLE of (**D**) NW, (**E**) NF powder; (**F**) Tr-PL curves.

X-ray diffraction (XRD) patterns of the ZnO NW and NF structured powders exhibited the signals belonging to the hexagonal wurtzite crystalline phase (space group: P63mc) (Fig. 1A). The crystallite sizes of NW and NF were calculated using the Scherrer equation as 29.7 nm and 13.4 nm, respectively. The diffraction peaks belonging to (100), (002), and (101) crystal planes have been chosen for the calculation. This outcome supports the previous report on ZnO morphologies produced in different pH conditions, in which an acidic medium led to the formation of ZnO with a smaller crystallite size^65^. Transmission electron microscopy (TEM) images of ZnO NW and NF are presented in Fig. 1B. Analysis of TEM images demonstrates that the average thickness and the length for ZnO NW are around 100 ± 20 nm and 900 ± 200 nm, respectively (Fig. 1B(a)). The outer surface of ZnO NW is relatively smooth. High-resolution TEM (HR-TEM) confirms the monocrystalline structure of NW, whereas TEM images reveal the lattice distance, d = 0.275 ± 0.05 nm corresponding to the (002) crystal plane of the ZnO Wurtzite phase. Figure 1B(b) shows the TEM images of ZnO NF. One can observe the ZnO "legs" with a variable thickness (tip thickness is around 20 nm, the middle thickness – 150–200 nm). The HR-TEM images demonstrate the polycrystalline structure of the investigated ZnO NF, where the average grain size has been calculated as 25 ± 12 nm. The lattice distance of ZnO grains was d = 0.8 ± 0.05 nm corresponding to the (002) crystal planes. It is seen that the ZnO NF has a more porous structure in comparison to ZnO NW. Besides, TEM analysis confirms that the morphology of ZnO NF corresponds to the more defective structure of the material. TEM images and their FFT (Figure S1 A–D) demonstrate that ZnO NW and NF have a monocrystalline and a polycrystalline nature, respectively.

Photoluminescence (PL) spectroscopy is an appropriate technique for determining the impurities or defects in nanoparticles^66–68^. Especially the effect of reaction conditions and annealing temperature on the defect-based emissions of hydrothermal grown ZnO can be interpreted by PL emissions in the visible region^69–71^. The PL spectra of ZnO NW and NF (Fig. 1C) demonstrate two emission PL bands. The characteristic small PL peak, centered in the UV range (383 nm, 3.24 eV), corresponds to the near band emission (NBE) that originates from exciton transition. The observation of higher NBE for ZnO NW is consistent with the literature that reported the basic medium during the synthesis of ZnO results in higher NBE PL emission^65^. The broad bands in the region between 400 and 900 nm are related to the defect level emission (DLE). A slight redshift in the center of these emission bands (631 for NF and 642 nm for NW) is associated with the grain growth and band-bending in ZnO nanocrystals. Moreover, the DLE band's intensity for ZnO NF is much smaller than for ZnO NW (Fig. 1C). One may conclude that this quenching in PL intensity of ZnO NF is due to the high concentration of the radiative recombination defects. Examining the DLE emission of both nanostructures, the main peak can be deconvoluted into three Gaussian peaks (Fig. 1D and E). The yellow emission (residing at ∼560 nm) is attributed to the presence of hydroxyl groups or Zn(OH)_2_, which are formed during annealing in air^68^. The orange-red emission located at ∼640–650 nm is usually credited for the presence of excess oxygen in ZnO, especially as a result of hydrothermal growth in an oxygen-rich environment^65,67^. The peak centered at ∼750 nm in the near-infrared (NIR) emission, which originates from the oxygen-related defects or zinc interstitials, is similar to those responsible for the red luminescence in ZnO^67,72^. Each contribution in emission for both ZnO NW and NF has been given in Fig. 1D and E, indicating the red luminescence is much higher in ZnO NW. The increase in orange-red and yellow emission from the transition from nanowire to nanoflower ZnO nanostructure might be attributed to the changes in OH^−^/H^+^ ratio which binds with oxygen due to the alteration of the pH value in the hydrothermal growth. Figure 1F shows the time-resolved PL (TR-PL) spectra for the DLE emission, obtained with an excitation wavelenghth of 337 nm and 3.5 ns pulse duration, indicating that the PL decay of ZnO NF is longer than that of NW powder.

**Figure 2:**
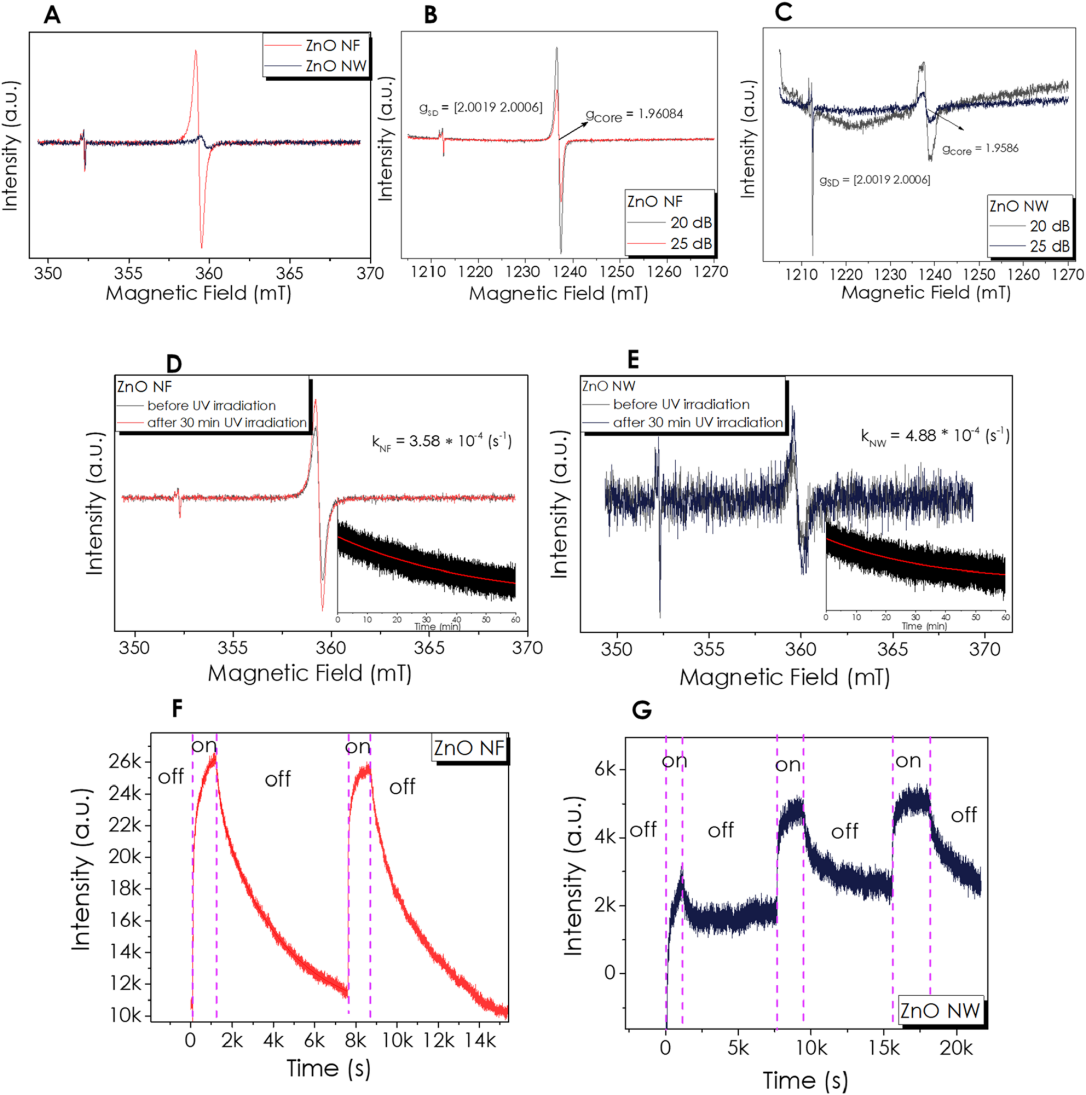
(**A**) X-band EPR spectra (9.879 GHz) of the ZnO-based materials, Q-band EPR spectra (33.984 GHz) of the NF **(B)**, and NW **(C)**, EPR spectra of the two in dark conditions (black line) and subsequent the irradiation time (red line) of (**D**) NF and (**E**) NW. The insets show the evolution of the EPR signal maximum after the UV-light excitation was stopped, EPR signal evolution during on–off UV-light cycles of (**F**) NF and (**G**) NW samples.

Electron paramagnetic resonance spectroscopy measurements were carried out at room temperature in X-band (Fig. 2A) on the two ZnO-based materials with different morphology, namely NF and NW. Both of them revealed the presence of two distinct EPR signals, consistent with results reported in previous works^73–75^. The first one in the lower magnetic field region stays the same for both morphologies, while the second one, which occurs at around 360 mT, changes in intensity and position (g-value). According to the power-sweep measurements result shown in Figure S2, Supporting Information, the observed resonances saturate differently, indicating that these signals are of different nature. The low field signals show the same saturation profile for both NF and NW, with a shallow saturation point at around 35 dB. The EPR signals of the NF and NW in the 360 mT region show no saturation at all, even at very low microwave attenuations (10 dB). The g-values of the EPR signals were calculated based on the Q-band measurements presented in Fig. 2B and C. The low field signal showed an axial g-value with g_SD_ = [2.0019 2.0006], which we associate based on the core–shell model^76,77^ to surface defects, also known as shallow-donor defects. The concentration, which is proportional to the EPR intensity of these paramagnetic centers, is approximately the same in both samples NF and NW. On the other hand, the EPR signals at around 3600 G have an isotropic g-value with g_NF_ = 1.96084 and g_NW_ = 1.9586. These paramagnetic centers are associated with ionized Oxygen or Zinc vacancies that appear in the ZnO crystal structure^78^. Based on the EDX evaluation explained later, these are more likely to be Zn vacancies than O vacancies. The Q-band measurements were carried out at two different microwave attenuations (25 and 20 dB) to avoid EPR signal saturation.

An equal amount of ZnO NF and NW samples were irradiated with UV-light (λ = 365 nm) for 30 min. Figure 2D and E show the EPR spectra of the two in dark conditions (black line) and subsequent the irradiation time (red line). The EPR signal intensity of the surface defects does not change after irradiation, indicating that the UV irradiation does not induce new defects on the surface of the materials. In contrast, the EPR signal intensity of the core-related paramagnetic centers increases significantly after UV irradiation in both cases. After irradiation, the EPR signal intensity at the maximum was monitored for relaxation for over 1 h (inset of Fig. 2D and E). Exponential decay of both ZnO NW and NW signals was observed but with different decay constants, which were calculated using the fitted t_1_ time parameter, with k = 1/t_1_. The obtained values for the decay constants k_ZnONF_ = 3.58 × 10^–4^ / s^−1^ and k_ZnONW_ = 4.88 × 10^–4^ / s^−1^ show that the photo-generated defects in the NW sample recombine faster than the ones in the NF sample. Further characterization has been performed via illuminating the samples with UV light in multiple on–off cycles. (Fig. 2F and G). These measurements show that the paramagnetic centers generated with UV-light in the ZnO NFs recombine almost entirely after 100 min, indicating an excellent recombination process, which remains the same after repeating the illumination process. On the other hand, the ZnO NW has a much more complex recombination process. The photo-generated paramagnetic centers do not recombine entirely after the first illumination cycle. This process becomes reversible only after the second cycle, indicating that two or more processes generate defects under illumination.

### Thin-film properties and rigid/flexible device assembly

**Figure 3:**
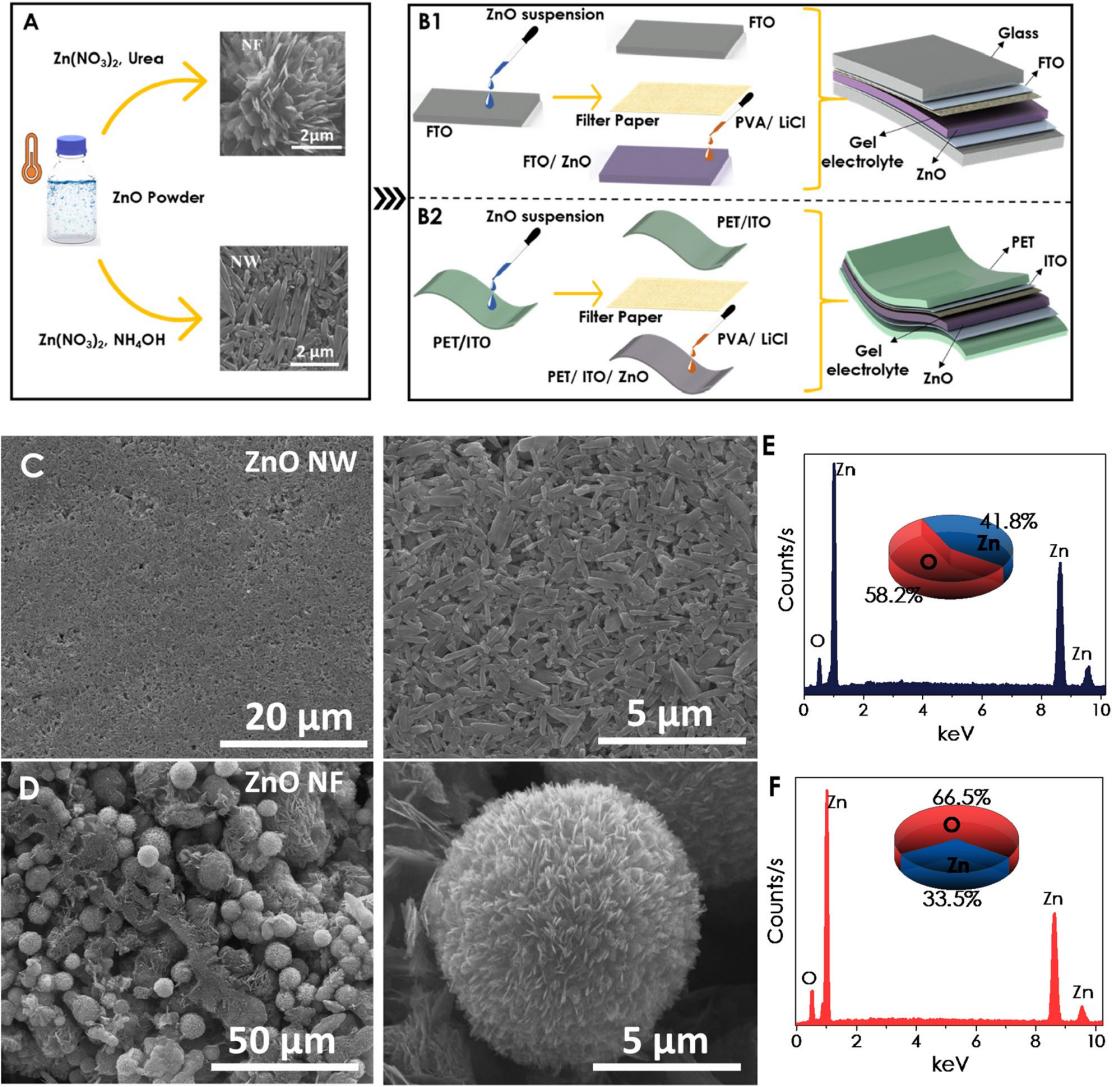
(**A**) Schematic demonstration for ZnO powder preparation, (**B**) the rigid and flexible P-SC assemble, respectively; ESEM images of ZnO (**C**) NW and (**D**) NF powder after spin-coated on FTO coated glass substrates; and EDX analysis results of ZnO (**E**) NW and (**F**) NF.

The nanostructured powder samples have been deposited on rigid and flexible substrates to fabricate a P-SC device, as shown schematically in Fig. 3A, B1 and B2. The applied assembly process is simple yet practical for preparing the P-SC. The SEM images in various magnifications have been depicted in Fig. 3C and D for ZnO NW and NF thin films prepared by the drop-casting technique. Morphological difference between the two powder samples has been revealed by the SEM images, in which ZnO NF thin film consists of micron-sized spheres, while ZnO NW thin film formed from one-dimensional wire-like structures. The elemental compositions of ZnO thin films in both morphologies have been determined by EDX as shown in Fig. 3E and F. The ZnO NW has a higher Zn% than the ZnO NF; both thin films were zinc deficient (inset Fig. 3E and F).

### Rigid devices' cyclic voltammogram (CV) and galvanostatic charge–discharge (GCD) performance

**Figure 4:**
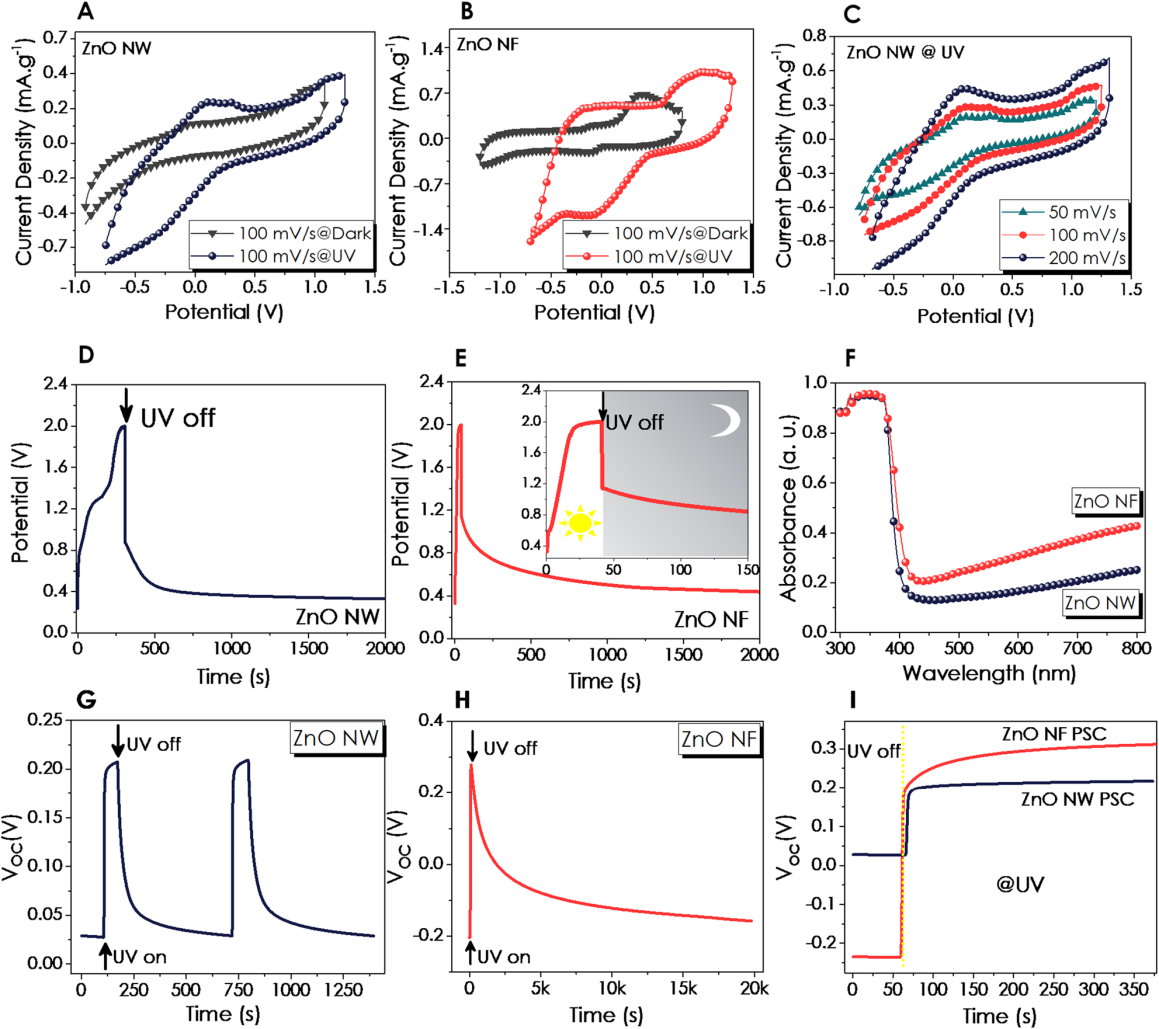
CV curves of **(A)** ZnO NW and **(B)** ZnO NF at dark and UV conditions, (**C**) ZnO NW at UV with various scan rates. The curves for photo-charging (at 0.55 mA g^−1^)/self-discharge in the dark under zero applied current after long-term GCD measurements; (**D**) ZnO NW, (**E**) ZnO NF, (**F**) UV–Vis Absorbance curves for both thin-film P-SC devices; Changes in open circuit potential (V_OC_) under UV illumination and dark conditions for (**G**) ZnO NW, (**H**) ZnO NF and (**I**) Comparison for V_OC_ at dark and under UV illumination for 5 min.

The CV curves in a potential range of − 1.0 to + 1.25 V and at a scan rate of 100 mV s^−1^ under UV illumination and dark conditions have been compared in Fig. 4A and B for ZnO NW and NF, respectively. The profile of the CV curves of the electrodes verified their pseudocapacitive nature^79^. The distinctive differences in the redox peak potentials, as increasing the CV curve's integral area under illumination, indicate the superior capacitance performance. Since the specific capacitance is proportional to the area under the CV curve, capacitance values have been calculated from CV data using the given equation (Equation S1) and tabulated in Table S1 (Supporting Information)^79,80^.

The specific capacitance of the ZnO NW-based P-SC has doubled with UV illumination. In contrast, the specific capacity of the ZnO NF device increased almost 2.4-fold, resulting in 1.98 and 4.15 mF g^−1^, respectively. The scan rate of the CV is the speed of scanning the applied potential. In faster scan rates, higher currents are observed due to a diminishing in the size of the diffusion layer^81^. The CV curves, recorded over various scan rates ranging from 50 to 200 mV s^−1^ (Fig. 4C), exhibited this expected enlargement in CV internal area as a consequence of the higher currents with an increase in the scan rates due to rapid diffusion of the ions in the electrolyte^82^. Besides, the CV profile of both photoelectrodes has been well preserved at higher scan rates, showing redox reversibility (Figure S3, Supporting Information). The disparities in the CV curves can be attributed to the variations in the morphology of the devices. The effect of morphological differences on CV measurements was demonstrated in a previous study comparing the flower and fiber-like morphologies of SnO_2_, in which the flower morphology was superior as compared to fiber SnO_2_^83^. Accordingly, the current response of ZnO NW is much lower than that of the ZnO NF structure based on the CV curves at dark and under UV light, possibly owing to the enhanced surface-to-volume ratio of the ZnO NF.

After a 3000-cycle test, the samples were charged at 1.1 mA g^−1^ under UV irradiation and discharged in the dark in an open-cell condition to understand the light effect better. The faster increase and lingering decrease in the potential upon UV on- and off- conditions have been observed for the P-SC-based ZnO NF compared to the ZnO NW-based P-SC device (Fig. 4D and E), possibly due to the relatively higher absorbance as presented in Fig. 4F. This is also consistent with the UV on–off cycled EPR spectra obtained for the ZnO NF powder sample given in Fig. 2F. Open-cell voltage (V_oc_) of the ZnO NW and NF-based P-SC has also been investigated (Fig. 4G–I). Although the V_oc_ of both the P-SC devices were similar under UV-illumination, after turning the light off, relaxation for ZnO NF-based P-SC to its initial condition took more than 5 h. On the other hand, ZnO NW P-SCs took around 500 s to relax. Although this result is not as evident as in V_oc_ measurements, it is compatible with PL and EPR results. The fact that the V_oc_ data was measured in the device configuration made the difference between NW and NF more pronounced.

**Figure 5:**
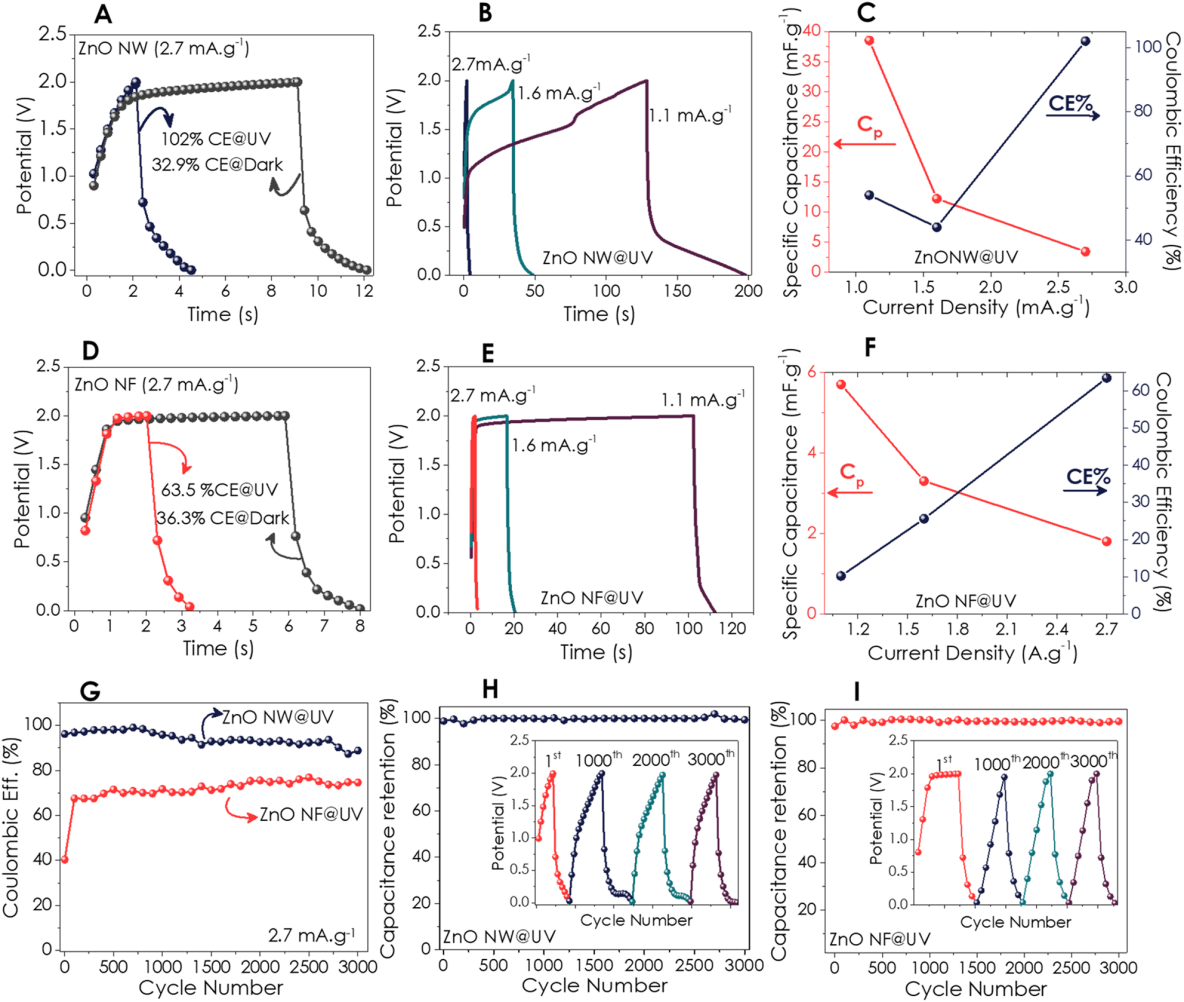
GCD curves; (**A**) comparison for ZnO NW under UV light and dark conditions at a 2.7 mA g^−1^ current density, (**B**) depending on changing current density under UV illumination, (**C**) capacitance and Coulombic efficiency, (**D**) comparison for ZnO NF under UV light and dark condition at 2.7 mA g^−1^ current density, (**E**) depending on changing current density under UV illumination, (**F**) capacitance and Coulombic efficiency, (**G**) Coulombic efficiency retention for 3000 cycles GCD under UV illumination. Capacitance retention and 1st, 1000th, 2000th and 3000th charge–discharge cycles (inset figure) for (**H**) ZnO NW and (**I)** ZnO NF.

The GCD curves have been obtained at various current densities under dark and UV-illumination (Fig. 5). Figure 4A depicts the GCD of the ZnO NW-based P-SC device of 2.7 mA g^−1^ under illuminated and dark conditions. It has been observed that the charging times of the P-SC devices illuminated with UV light are reduced approximately 5 times compared to those operating in the dark. Hence, Coulombic efficiencies (CE%) have increased due to faster charging under the effect of light. The CE% of the ZnO NW-based P-SC increased from ~ 33 to 102% under illumination (Fig. 5A). The positive impact of UV light has been observed in the GCD plots, which have been measured in the current densities range of 1.1 to 1.6 mA g^−1^ (Fig. 5B). In addition, the calculated capacitance-CE% data over the changing current density under UV excitation has been displayed in Fig. 4C, showing the best Coulombic efficiency at the current density of 2.7 mA g^−1^. In contrast, 39.4 mF g^−1^ of specific capacitance has been calculated for the current density of 1.1 mA g^−1^. Comparing the GCD measurements obtained for ZnO NW and NF in the dark and under UV illumination with low current density (0.55 mA g^−1^) (Figure S4, Supporting Information) exhibited an outstanding enhancement by increasing the discharge time under UV. The GCD curves with varying current density at dark conditions have been given in Supporting Information (Figure S5, Supporting Information).

On the other hand, the GCD curves of the ZnO NF-based photo-supercapacitor device have displayed a shorter charging-discharge time resulting in lower overall CE% for both dark and illuminated compared to the ZnO NW (Fig. 5D–E). In Fig. 5F, the CE% and specific capacitance values have been much less than that of ZnO NW due to the shortening discharge time. The capacitance data at low current density (1.1 mA g^−1^) have been used to determine the energy density as 78.1 and 11.4 mWh kg^−1^ for NW ad NF, respectively. Furthermore, the CE% of NF increased approximately 1.8-fold after the 2nd GCD-cycle (Fig. 5H). Then again, CE% of NW was higher than 90% almost for 1400-cycle and decreased to 80% after 3000-cycle (Fig. 5H). Capacitance retention from cycling performance which was carried out at 2.7 mA g^−1^ over the 3000 cycles under UV illumination, has been displayed in Fig. 5H and I. The curves of 1st, 1000th, 2000th, and 3000th cycles with triangular-like shapes given as inset of figures of Fig. 5D–E confirmed the stability of both ZnO NW and NF P-SC devices. Eventually, capacitance retention data over 3000 cycles for both ZnO-based devices confirmed excellent long-term cycling stability.

### CV and GCD performance of flexible devices

**Figure 6:**
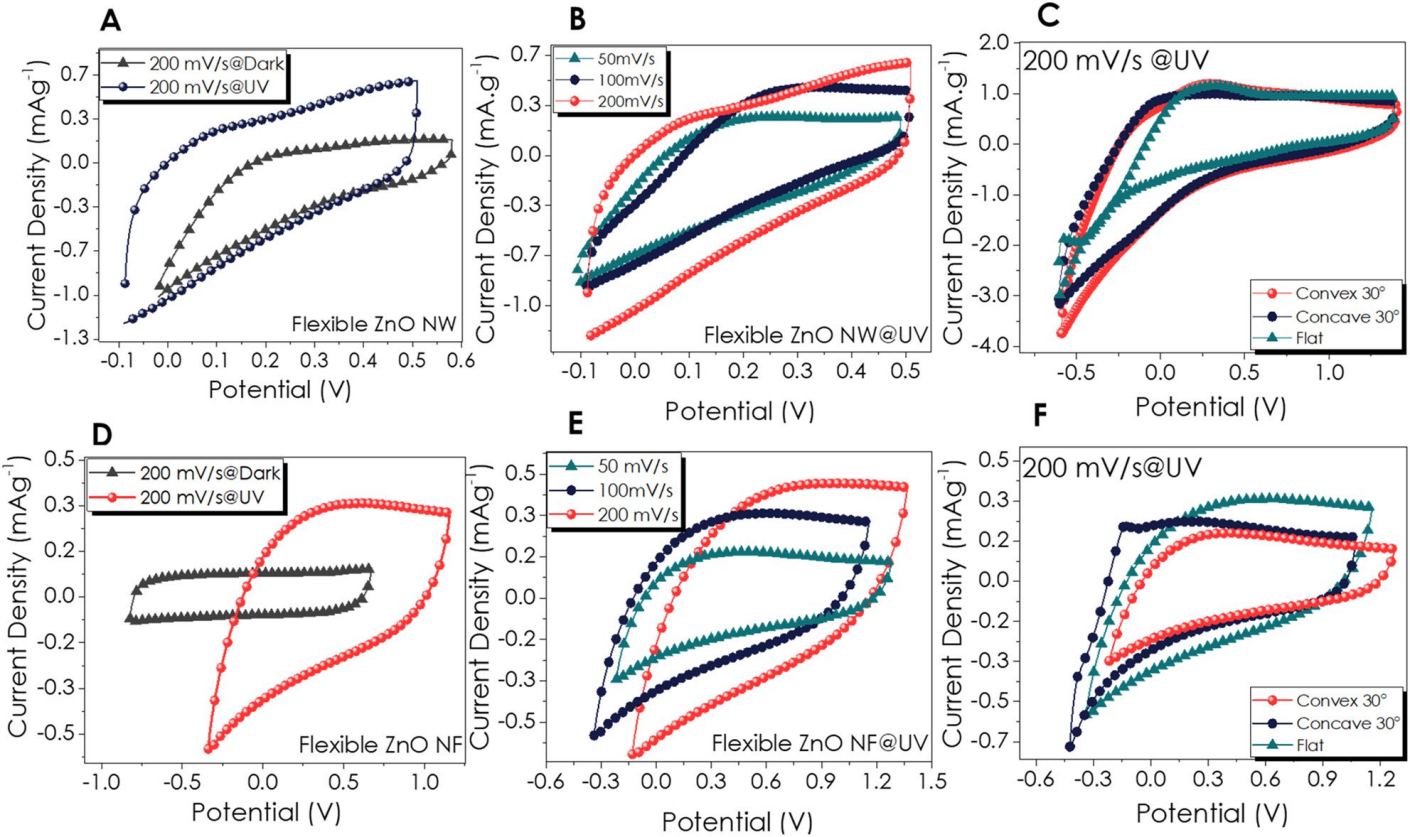
CV curves of flexible ZnO NW based P-SC; (**A**) with 200 mV s^−1^ scan rate under UV and dark conditions, (**B**) under UV light with different scan rates, (**C**) bent and flat P-SC with 200 mV s^−1^ scan rate; and flexible ZnO NF based P-SC; (**D**) with 200 mV s^−1^ scan rate under UV and dark conditions, (**E**) under UV light with different scan rates, (**F**) bent and flat P-SC with 200 mV s^−1^ scan rate.

The CV curves of the flexible ZnO P-SC devices are given in Fig. 6. In Fig. 6A, an unprecedented increase in the CV area of the ZnO NW P-SC device under UV illumination has been observed at a scan rate of 200 mV s^−1^ concerning the dark condition. Figure 6B shows the CV curve taken under UV illumination with different scan rates indicating the quasi-rectangle shapes instead of redox peaks. Further, bending of the P-SC device in a concave and convex orientation at a 30° angle enhanced the CV curve, possibly due to the increased active area after bending (Fig. 6C). On the other hand, an excellent increase in the CV area after UV illumination for flexible ZnO NF P-SC (Fig. 6D). CV curves of ZnO NF P-SC at different scan rates have quasi-rectangle shapes, the same as ZnO NW P-SC (Fig. 6E). The CV curves were measured upon bending experiments (concave-convex orientations with 30°), in which the concave bending exhibited better performance than convex bending (Fig. 6F). CV and GCD curves of flexible ZnO NW and NF at dark have been given in Figure S6, Supporting Information. Flexible NW and NF-based P-SC had similar energy and power densities (Figure S7A, Supporting Information).

**Figure 7:**
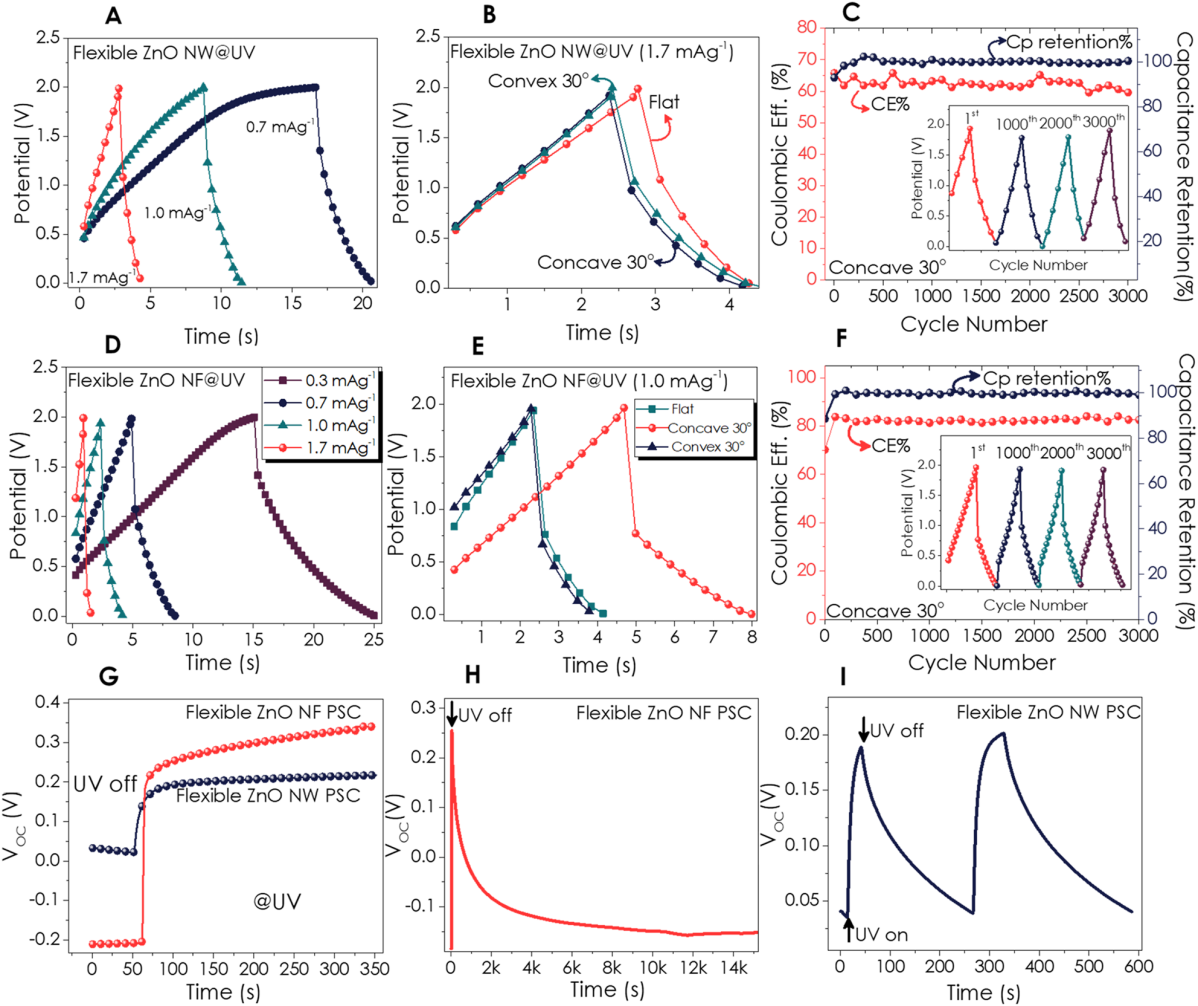
GCD curves for Flexible ZnO NW P-SCs; (**A**) at various current densities under UV light, (**B**) comparison of the GCD curves of flat and bent P-SC, (**C**) CE% and capacitance retention of concave P-SC, GCD curves for Flexible ZnO NF; (**D**) at various current densities under UV light, (**E**) comparison GCD curves of flat and bent P-SC, (**F**) CE% and capacitance retention of concave P-SC; Open circuit potential (V_OC_) curves; (**G**) at dark and under UV illumination for 5 min, V_OC_ response to chopped light and relaxation duration for flexible (**H**) ZnO NF and (**I**) NW P-SC devices.

GCD measurements have been carried out at a fixed potential of 2 V and various current densities. Figure 7A presents the GCD curves at the current densities ranging from 0.7 to 1.7 mA g^−1^. As for the current density of 1.0 mA g^−1^, the Cp and Eg values have been calculated as 1.46 mF g^−1^ and 2.92 mWh kg^−1^, respectively. The GCD curves (Fig. 7B) presented similar behavior upon the bending experiment, but bending in a concave orientation with 30° resulted in slightly higher Cp as 1.8 mF g^−1^ (for 1.7 mA g^−1^). At lower current densities, CE% dropped from ~ 70% to 59% and 27% for 1.0 and 0.7 mA g^−1^ respectively. However, the Cp values have increased (1.9 and 2.3 mF g^−1^ for 1.0 and 0.7 mA g^−1^, respectively). Therefore, we decided to conduct a long-term cycle of GCD experiments for 1.0 mA g^−1^ (Fig. 7C). The CE% and Cp retention for 3000 cycles have been given in Fig. 6C along with single cycles at chosen intervals (inset of Fig. 7C). On the other hand, flexible ZnO NF P-SC exhibited shorted charge–discharge time (almost half of ZnO NW), as shown in Fig. 7D for each current density.

The bending of ZnO NF P-SC at 30° concave orientation (Fig. 7E) resulted in higher Cp values (1.65 mF g^−1^ for 1.0 mA g^−1^). Figure 7F exhibits the CE% and Cp retention for NF P-SC device for 3000 cycles indicating good stability of the device. Consequently, the Eg value increased from 2.9 to 3.6 mWh kg^−1^ for ZnO NW and 1.8 to 3.6 mWh kg^−1^ for NF after bending. Finally, the flexible design showed that both nanostructures have piezoelectric properties^84,85^, which resulted in an increase in CE% and energy density after bending. It is known that ZnO is a preferable material for piezoelectric nanogenerator applications which rely on the conversion of mechanical energy into electrical energy^86–88^. For instance, Xu et al. have first reported that the vertical and lateral integration of ZnO nanowires could generate sufficient power to operate real devices^89^. Almost a decade later, ZnO nanosheets have been demonstrated as piezoelectric two-dimensional DC generators^87^. The P-SC performance of the rigid and flexible ZnO NF and NW-based devices have been tabulated for a clear comparison (Table 1). The changes in V_OC_ under UV illumination and relaxation behavior under dark conditions for both flexible ZnO P-SC devices have been given in Fig. 7G–I. ZnO NF exhibited drastic V_OC_ increases under UV illumination as compared to ZnO NW (Fig. 7G). After chopping the UV light, the relaxation time was much longer for this device, the same as for the rigid ZnO NF P-SC presented in the previous section as compared to the rigid and flexible ZnO NW.

**Table 1:** Comparison of P-SC performance of rigid and flexible ZnO-based devices.

Device	Current Density (mA g^−1^)	t_d_ (s)	Cp (mF g^−1^)	E (mWh kg^−1^)	C_retention_ (%)
ZnO NW@UV rigid	1.1	71.0	39.1	78.1	98.9
ZnO NW@UV flexible*	1.0	3.5	1.8	3.5	99.8
ZnO NF@UV rigid	1.1	10.4	5.7	11.5	99.1
ZnO NF@UV flexible*	1.0	3.3	1.7	3.3	99.4

*P-SC measurements of flexible devices were obtained at 30° concave bending.

### Electrochemical impedance spectroscopy of the devices

**Figure 8:**
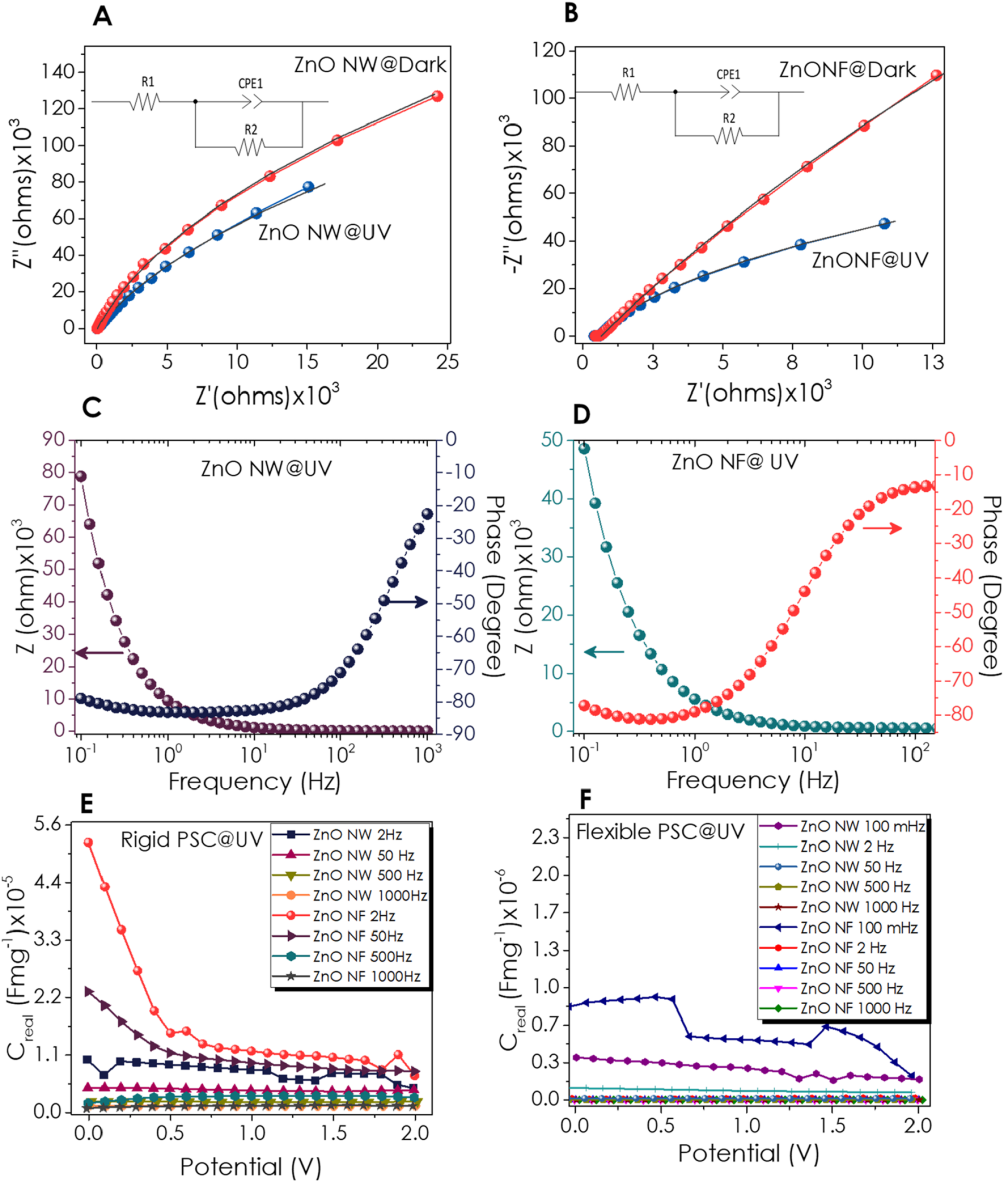
EIS measurements were performed in the scan range: 1 MHz to 0.1 Hz at 0 V. Nyquist Plot; (**A**) ZnO NW, (**B**) ZnO NF (The symbols stand for experimental data; the solid lines are the curves fitted to the electrical circuit model depicted in the inset), Bode plots (**C**) ZnO NW and **(D)** ZnO NF, Areal Capacitance at various frequencies at UV light **(E)** ZnO NW, **(F)** ZnO NF.

Electrochemical impedance spectroscopy (EIS) measurements have been performed to analyze the electrochemical characteristics of the ZnO NW and NF electrode/electrolyte interface at applied bias potentials of 0 V within the scan range from 1 MHz to 0.1 Hz. The typical Nyquist plots of ZnO nanostructured electrodes have been presented in Fig. 8A and B. A similar Nyquist feature for both nanostructures has been observed in the dark and under UV illumination. The low semicircle indicates the Faradaic charge transfer between electrodes and electrolyte. The Nyquist plot diameters of the supercapacitors are less under UV illumination than those in dark conditions, meaning less charge transfer resistance and easier ion diffusion in a redox reaction. The Nyquist plots of both photo supercapacitor devices have been well fitted with an equivalent circuit given as the inset of Fig. 8A–B, indicating the aptness of the proposed equivalent circuit model. The tabulated EIS parameters have been shown in Table 2. *R*_1_ represents the electrolyte's bulk resistance, while R_2_ represents the charge transfer resistance. The sum of *R*_1_ and R_2_ is the total resistance which defines the equivalent series resistance (ESR), which was higher for ZnO NF-based P-SC device. On the other hand, both P-SC devices' CPE values have been increased under UV illumination.

**Table 2:** Parameters of the equivalent circuit model, derived from fitting to EIS data.

Device	R_1_(Ω)	R_2_(MΩ)	Q_2_(F.s^a−1^) × 10^6^	a_2_
ZnO NW@Dark	34.5	1.29	12.0	0.951
ZnO NW@UV	32.6	0.78	18.8	0.936
ZnO NF@Dark	510.3	18.24	14.5	0.922
ZnO NF@UV	502.6	1.30	33.3	0.916

Bode plots of phase angle changes with frequency for ZnO-based asymmetric P-SCs under UV light (Fig. 8C–D) and dark conditions (Figure S8, Supporting Information). In the Bode phase plot, the phase shift approaches ~  − 80° in the low-frequency region, showing that both ZnO electrodes accomplish more like capacitors with the diffusion process. At UV illumination, the phase angle of ZnO NW and NF reaches − 45° at a capacitor response frequency of 267 and 9.57 Hz, respectively. Figures 8E and F show the frequency-dependent capacitance versus voltage curves for rigid and flexible substrates under UV illumination, respectively. For the NW-based rigid P-SC devices, variation of the real component of the capacitance is nearly independent of the applied voltage at frequencies ranging from 2 Hz to 1 kHz (Fig. 8E). Moreover, the capacitance of the NW-rigid devices decreased with increasing frequency. The maximum specific capacitance of 1.1 μFg^−1^ has been observed for 0 V applied bias and a frequency of 2 Hz for the NW-rigid P-SC. Although the high-frequency behavior of the NF-rigid device was similar to NW-rigid P-SC, at 2 Hz and 0 V bias, the specific capacitance of the NF-rigid was increased and reached 5.3 μFg^−1^. A similar voltage and frequency dependency of flexible P-SC has been observed. However, lower specific capacitance values have been calculated for NW and NF-based flexible devices. The difference between NF and NW-based devices in the low-frequency range can be attributed to the lower relaxation time due to the more pronounced defect levels observed from EPR and PL analysis. The same tendency has been obtained for the flexible P-SC with lower capacitance values than the rigid design (Fig. 8F). Frequency and applied bias voltage-dependent real capacitance plots ZnO NW and NF at dark, showing similar tendency with illuminated samples, have been given in Supporting Information (Figure S9, Supporting Information).

## Conclusion

In this study, the drop-casting technique was successfully used to integrate NW and NF ZnO powders into P-SC devices. Compared to the complex P-SC structures reported in the literature, simpler rigid and flexible P-SCs have been developed here. Besides, the performance relation with defects in ZnO powders has been established via EPR supported by TEM and PL. Our findings show that the ZnO NF structure has more core defects than the NW powders, resulting in lower Coulombic efficiency (CE%) and energy density. Although the dark CE% of both NW and NF were comparable (33% and 36%, respectively), after illumination with UV light, the CE% of NW and NF increased 3 and 1.8 fold and reached 102 and 64%, respectively. Since the excess charges created by the UV irradiation provide the extension of the discharge time of the capacitor, a significant increase in CE% has been detected. The energy density of 78.1 mWh kg^−1^ obtained for the NW-based P-SCs at 1.1 mA g^−1^ current density under UV illumination is one of the highest reported values in the literature among the only metal oxide-based electrodes (Figure S7B, Supporting Information). It is also essential to emphasize that our device has only ZnO powders and does not contain any other carbon or metal-based material. Therefore, building heterostructured hybrid P-SC electrodes can enhance energy and power density. Last but not least, capacitance retention of both NW and NF-based P-SC was about 100% after 3000-cycle showing the very high stability of the device. Given these findings, we can conclude that defect control is crucial for ZnO-based nanomaterials to be adopted into highly efficient flexible and rigid P-SC applications.

## Materials and methods

### Materials

Zinc nitrate hexahydrate (Zn(NO_3_)_2_6H_2_O, ACS reagent, ≥ 98%), urea (CH_4_N_2_O, ACS reagent, 99.0–100.5%), nitric acid (HNO_3_, 99.8–100.5%), ammonium hydroxide (NH_4_OH, ≥ 85%), were purchased from Sigma-Aldrich for hydrothermal growth of ZnO NF and NW nanostructures. Polyvinyl alcohol (PVA) and lithium chloride (LiCl, ACS reagent) has been used as gel electrolyte components. Fluorine-doped tin oxide-coated glass (FTO, resistivity 10 Ω /square) has been purchased from Sigma-Aldrich.

### Synthesis of ZnO powder

ZnO powder has been synthesized based on the procedure which was reported in our previous work^59^. Briefly, a hydrothermal reaction solution to synthesize NF powder has been prepared from the 100 ml aqueous solution of 0.05 M Zn(NO_3_)_2_·6H_2_O and 1.0 M urea with the pH value adjusted to 5.4 using concentrated HNO_3_. The reaction solution was kept in a standard reaction oven at 80 °C for 3 h. After the reaction has been completed, the powder product has been filtrated and washed with de-ionized (DI) water and ethanol, followed by calcination at 300 °C for 30 min. ZnO NW powder has been synthesized by applying the same procedure using 0.1 M Zn(NO_3_)_2_·6H_2_O and 0.15 M NH_4_OH at 80 °C for 3 h, followed by a calcination step at 300 °C for 30 min. The precursor, medium pH, and reaction duration variation alter the morphological variations of ZnO powder.

### Fabrication of the solid-state P-SC devices

The ZnO nanopowders have been coated onto fluorine-doped tin oxide (FTO) coated glass via drop-casting to form photo-active electrodes. PVA/LiCl gel was used as a solid electrolyte. Bare FTO coated glass has been used as received as a non-photoactive electrode. Flexible P-SC has been deposited on indium-doped tin oxide (ITO) coated PET substrates applying the same procedure described above. The detail of the experimental procedure, characterization, and electrochemical measurements have been given in Supporting Information.

## Supplementary Information

Supplementary Information.
