# Author Correction: Photo-supercapacitors based on nanoscaled ZnO

**DOI:** 10.1038/s41598-023-35687-3

**Published:** 2023-05-25

**Authors:** Cigdem Tuc Altaf, Ozlem Coskun, Alihan Kumtepe, Arpad Mihai Rostas, Igor Iatsunskyi, Emerson Coy, Emre Erdem, Mehmet Sankir, Nurdan Demirci Sankir

**Affiliations:** 1 Department of Materials Science and Nanotechnology Engineering, TOBB University of Economics and Technology, Sogutozu Caddesi No 43 Sogutozu, 06560 Ankara, Turkey; 2 Micro and Nanotechnology Graduate Program, TOBB University of Economics and Technology, Sogutozu Caddesi No 43 Sogutozu, 06560 Ankara, Turkey; 3 National Institute for Research and Development of Isotopic and Molecular Technologies, 67-103 Donat, PO 5 Box 700, 400293 Cluj-Napoca, Romania; 4 NanoBioMedical Centre, Adam Mickiewicz University in Poznań, Wszechnicy Piastowskiej 3, 61-614 Poznań, Poland; 5 Faculty of Engineering and Natural Sciences, Sabanci University, Orhanli, 34956 Tuzla, Istanbul Turkey

Correction to: *Scientific Reports* 10.1038/s41598-022-15180-z, published online 07 July 2022

The original version of this Article contained an error in the Acknowledgements section.

“I.I. acknowledges the partial financial support from the SONATA BIS project 2020/38/E/ST5/00176.”

now reads:

“I.I. was partly funded by the NCN SONATA-BIS Program (UMO-2020/38/E/ST5/00176).”

The original Article has been corrected.

