# Supplementary material for: Photo-supercapacitors based on nanoscaled ZnO
Source: Sci Rep. 2022 Jul 7;12:11487. doi: 10.1038/s41598-022-15180-z (PMC9262889; doi:10.1038/s41598-022-15180-z)
Supplement: Supplementary file 1 — Supplementary Information. [file 41598_2022_15180_MOESM1_ESM.pdf]

Supporting Information

Photo-Supercapacitors Based on Nanoscaled ZnO

Cigdem Tuc Altaf,[a] Ozlem Coskun,[b] Alihan Kumtepe,[b] Arpad Mihai Rostas,[c] Igor Iatsunskyi,[d] Emerson Coy,[d] Emre Erdem,[e] Mehmet Sankir,[a, b] † Nurdan Demirci Sankir[a, b] ‡

[a] Department of Materials Science and Nanotechnology Engineering, TOBB University of Economics and Technology, Sogutozu Caddesi No 43 Sogutozu 06560 Ankara, Turkey

[b] Micro and Nanotechnology Graduate Program, TOBB University of Economics and Technology, Sogutozu Caddesi No 43 Sogutozu 06560 Ankara, Turkey

[c] National Institute for Research and Development of Isotopic and Molecular Technologies, 67-103 Donat, PO 5 Box 700, 400293 Cluj-Napoca, România

[d] NanoBioMedical Centre, Adam Mickiewicz University in Poznań, Wszechnicy Piastowskiej 3, 61-614, Poznań, Poland

[e] Faculty of Engineering and Natural Sciences, Sabanci University, Orhanli, Tuzla, 34956, Istanbul, Turkey

Corresponding author email: † msankir@etu.edu.tr, ‡ nsankir@etu.edu.tr

Device Fabrication and Characterizations

The synthesized nanoparticles (0.1 g) have been dispersed in an isopropanol/1-butanol/methanol mixture (3/1/0.5 volume ratio) in an ultrasonic bath for 10 min. The dispersion has been spun on FTO at 1000rpm / 30 s three times, followed by drop-casting to achieve the desired amount of active material on the surface. ZnO nanoparticles have assembled the P-SCs coated electrode (1.5 cm x 1.5 cm), and bare FTO coated glass substrates with filter paper sandwiched in between as a separator. PVA / LiCl gel was used as a solid electrolyte. According to a previously reported procedure, PVA / LiCl gel electrolyte has been prepared.[1] Briefly, 3 g PVA was mixed with 30 mL LiCl (5 M) aqueous solution and heated at 85 °C for 1 h under vigorous stirring. The electrodes and the separator were dipped in the PVA/LiCl solution, solidified, and kept at room temperature overnight. Flexible P-SC has been deposited on indium-doped tin oxide (ITO) coated PET substrates applying the same procedure described above.

The morphological and structural characterizations have been performed by Scanning Electron Microscopy (SEM, FEI, Quanta 200 FEG instrument), transmission electron microscopy (TEM) (JEOL ARM 200F) high-resolution transmission electron microscope (200 kV) with an Energy Dispersive X-ray (EDX) analyzer. Photoluminescence (PL) of ZnO NW and NF was generated by a HeCd laser (325 nm) and the emission spectra were recorded in the range 350-1100 nm by Ocean Optics Spectrometer QE65. The time-resolved PL (TR-PL) tests for DLE emission were performed using an NL100 nitrogen laser (Stanford research) as an excitation source. The excitation wavelength is 337 nm, and the pulse duration is 3.5 ns. The pulse energy is 170 μJ, which results in a peak power of 45 kW and average power of 3 mW. As a synchronized detector, a multi-alkali amplified PMT (Thorlabs) operating in the range 230 - 920 nm was used. Figure 1F shows TR-PL spectra indicating that the PL decay of ZnO NF is longer than that of NW powder.

The X and Q-band Electron Paramagnetic Resonance (EPR) spectroscopy measurements were carried out with a continuous-wave Elexsys 500 EPR spectrometer (Bruker AXS GmbH, Karlsruhe, Germany) equipped with a Bruker X-SHQ 4119HS-W1 X-Band resonator and a WT-Q. The UV irradiation of the samples for the EPR measurements was carried out with an M365FP1 - 365 nm Fiber-Coupled LED from Thorlabs. Optical properties have been investigated using a PerkinElmer UV − vis spectrometer Lambda2S (200 − 1100 nm). The Electrochemical Impedance Spectroscopy (EIS) measurements have been performed at the potential of 0V over the frequency range from 1MHz to 0.1 Hz using a Gamry workstation under dark and UV light. The performance evaluations of P-SC have been investigated by electrochemical characterization methods such as Cyclic Voltammetry (CV), and galvanostatic charge/discharge (GCD) in a two-electrode configuration system by assembling the supercapacitor electrodes in Gamry potentiostat/galvanostat workstation under dark and UV illumination. A UV radiation source (8.8 $mW.cm^{-2}$) has been used for the study of UV-irradiation-dependent electrochemical measurements. Coulombic efficiency (%), specific capacitance (Cp, $F.g^{-1}$), specific energy (E, $Wh.kg^{-1}$), and specific power (P, $W.kg^{-1}$) of the supercapacitor device have been evaluated from the equations given in the supporting information.

Calculation of GCD and CV performance

Specific capacitance (C) has been calculated from CV data using the following equation[2].

$$C = \frac{1}{2mkV}\int_{V^-}^{V^+} I(V)dV \quad (1)$$

where C ($F.g^{-1}$) denotes the specific capacitance, while V (V) corresponds to the potential window, I (A) represents the discharge current, and m (g) is the mass of the active material, and k ($V.s^{-1}$) is scan rate.

The discharge section of the GCD curves could be applied to evaluate Cp from Equation (1).[3]

$$C_p = \frac{It_d}{m\Delta V} \;\; (F.g^{-1}) \qquad (2)$$

Moreover, by the Cs values, E and P of the supercapacitor cell can be evaluated as;[4]

$$E = \frac{C_p \Delta V^2}{2} \;\; (Wh.kg^{-1}) \quad (3)$$

$$P = \frac{E}{t_d} \; (W.kg^{-1}) \qquad (4)$$

where Cs is the specific capacitance of the electrode, t_d is time (s) of discharge, t_c is time (s) of charging, I is the discharge constant current (A), m is the mass of material (g), and ΔV is the potential window (V).

Finally, Coulombic Efficiency (CE%) can be evaluated from the following equation;

$$CE(\%) = \frac{\Delta t_d}{\Delta t_c} x100 \qquad (5)$$

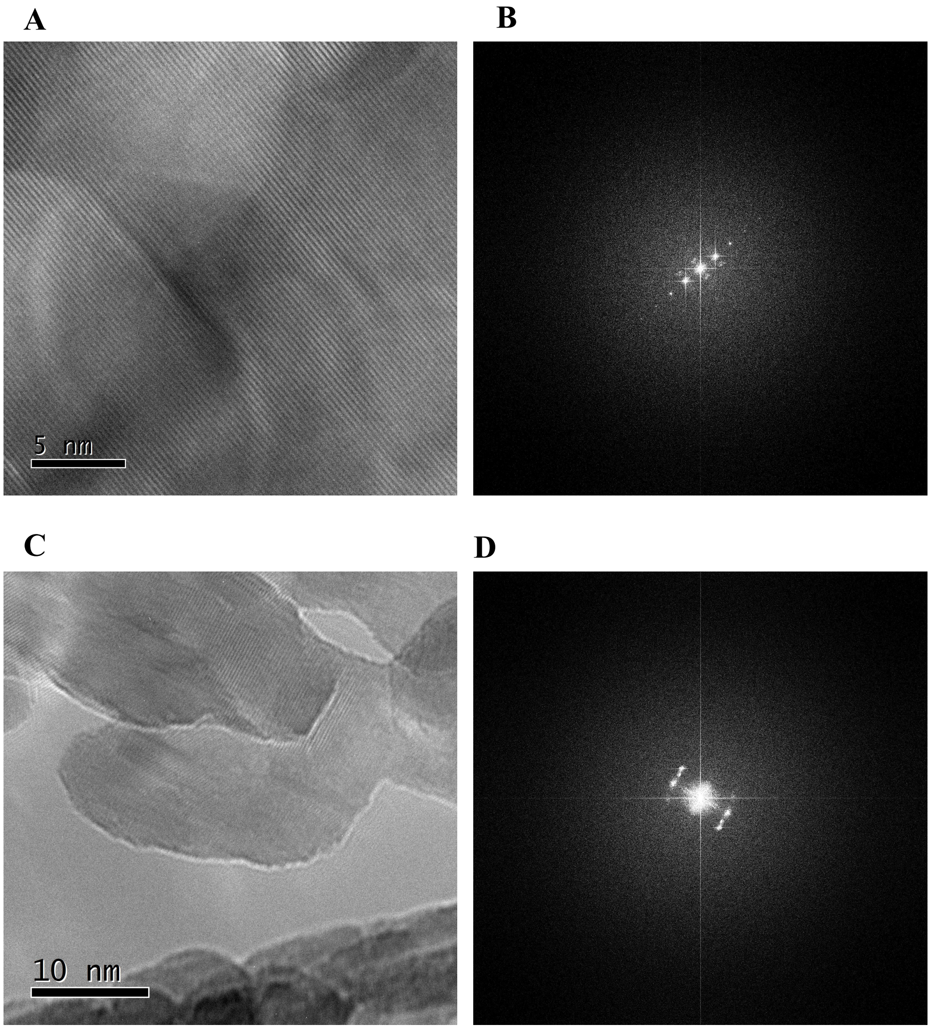

Figure S1. (A) HR-TEM image and its (B) FFT for ZnO NW, (C) HR-TEM image and its (D) FFT for ZnO NF

Electron Paramagnetic Resonance Spectroscopy Power Sweep Measurements

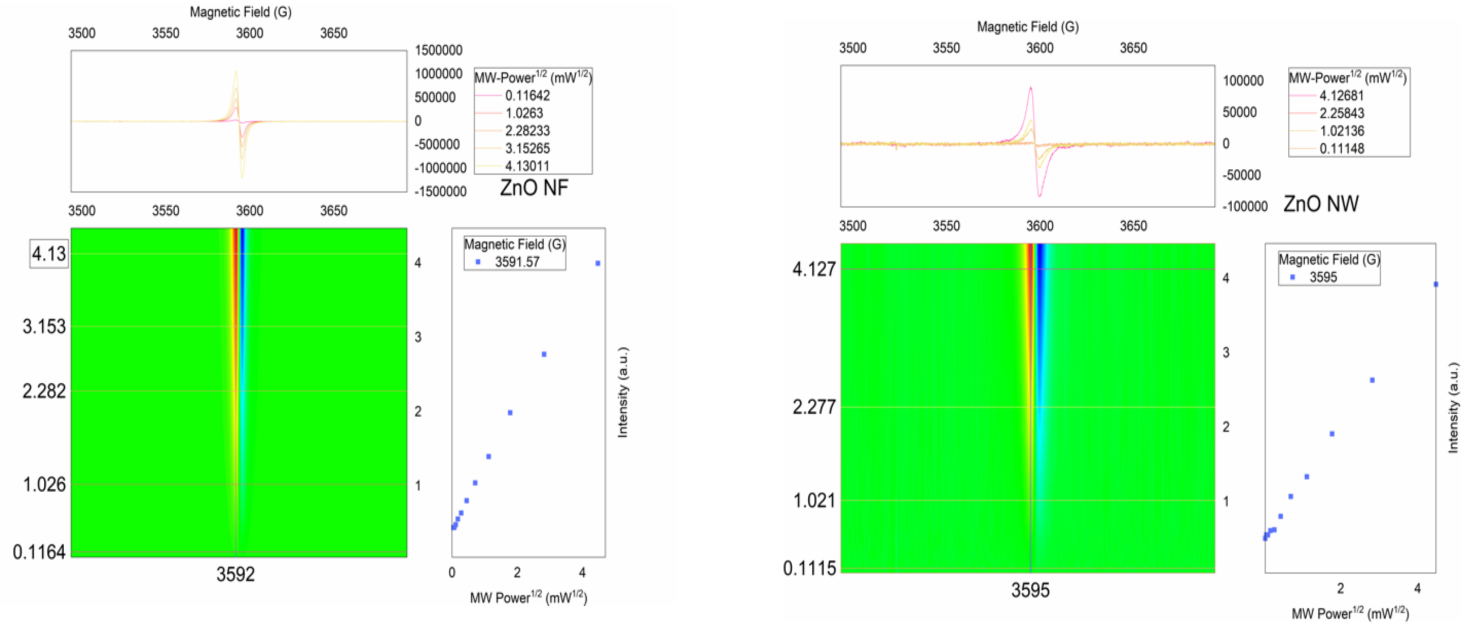

Figure S2. Power sweep measurements were carried out on the ZnO-NF and ZnO-NW samples in X-band at room temperature.

Performance Summary

Table S1 Specific capacitance values calculated from CV scan at 100mV/s of scan rate.

Device	Area (AV)	Scan rate (V/)	Potential window (V)	Mass (g)	C_p(mF/g)	E (mWh.kg^{-1})
ZnONW@Dark	7.22E-06	0.1	1.99	0.018	1.00	2.0
ZnONW@UV	1.42E-05	0.1	1.99	0.018	1.98	3.8
ZnONF@Dark	1.21E-05	0.1	1.99	0.018	1.69	3.4
ZnONF@UV	2.98E-05	0.1	1.99	0.018	4.15	8.3

Cyclic voltammogram

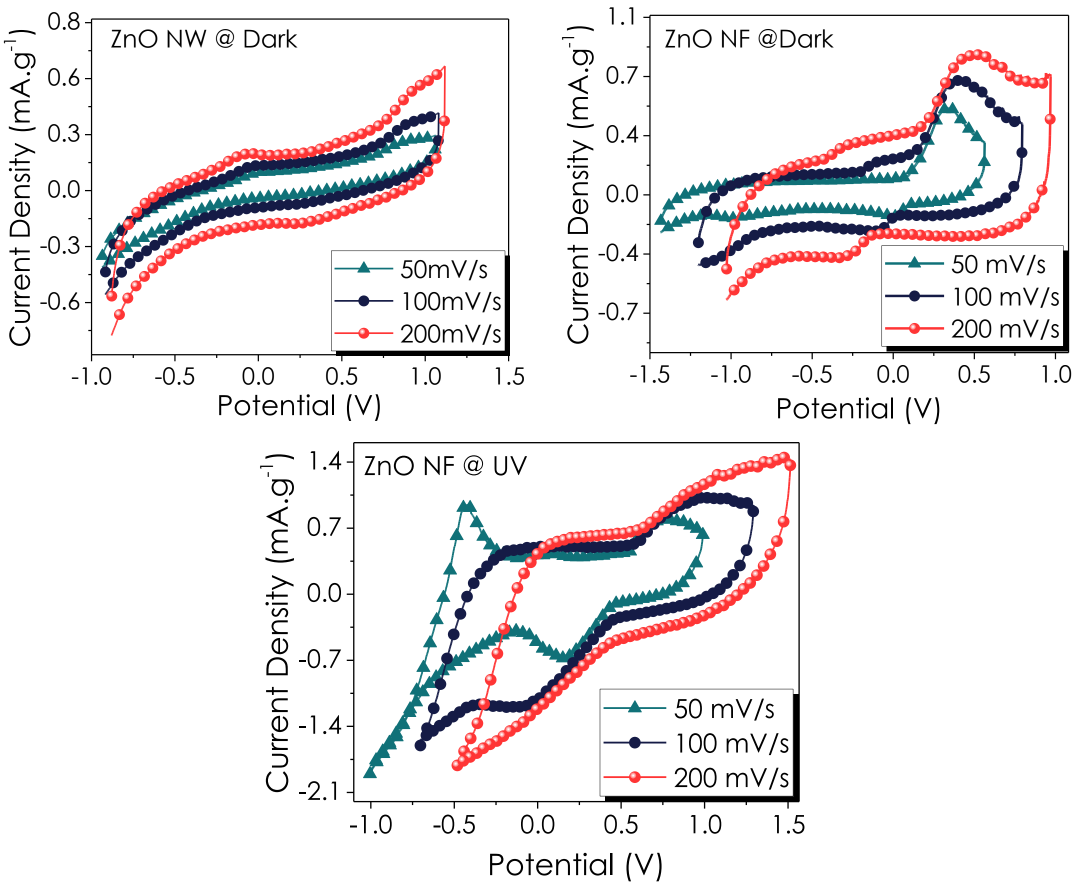

Figure S3. CV curves at dark and under illumination

Galvanostatic charge-discharge (GCD) curves

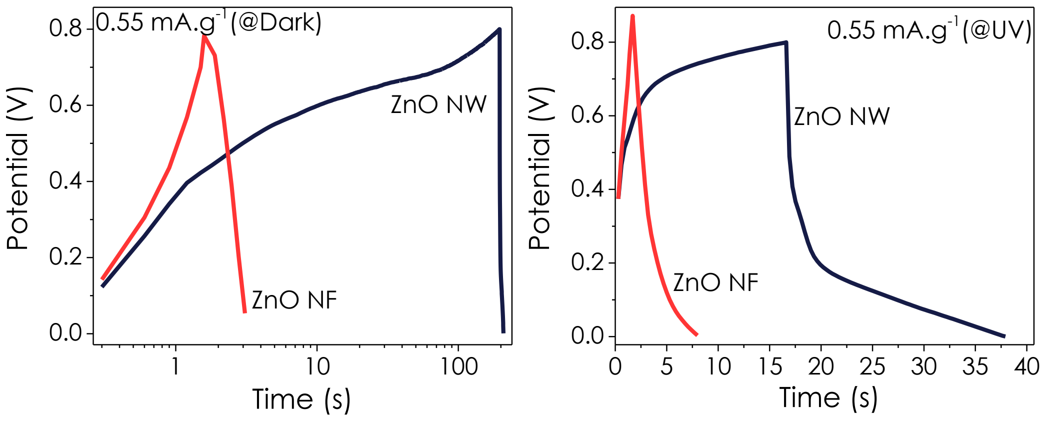

Figure S4. GCD curves at 0.55 $mA.g^{-1}$ current density

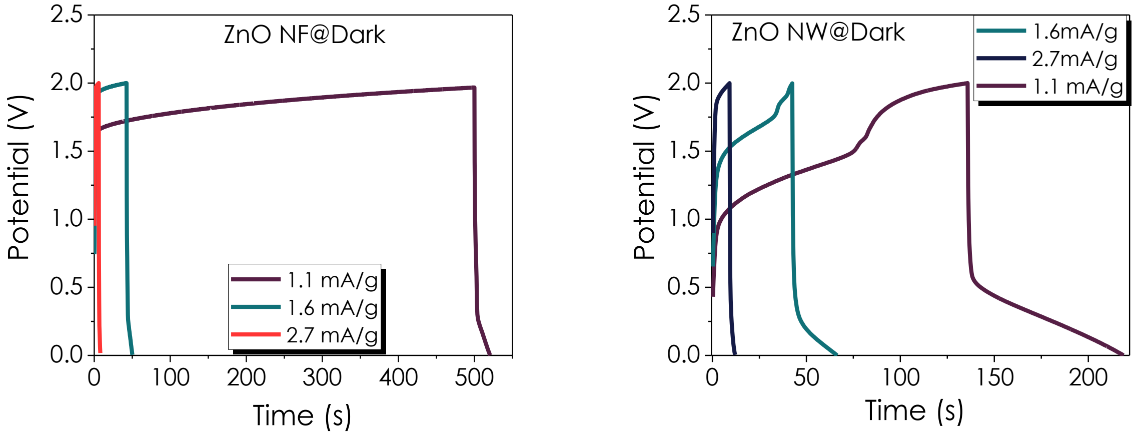

Figure S5. GCD curves with varying current density in dark condition

Flexible ZnO NW and NF P-SC

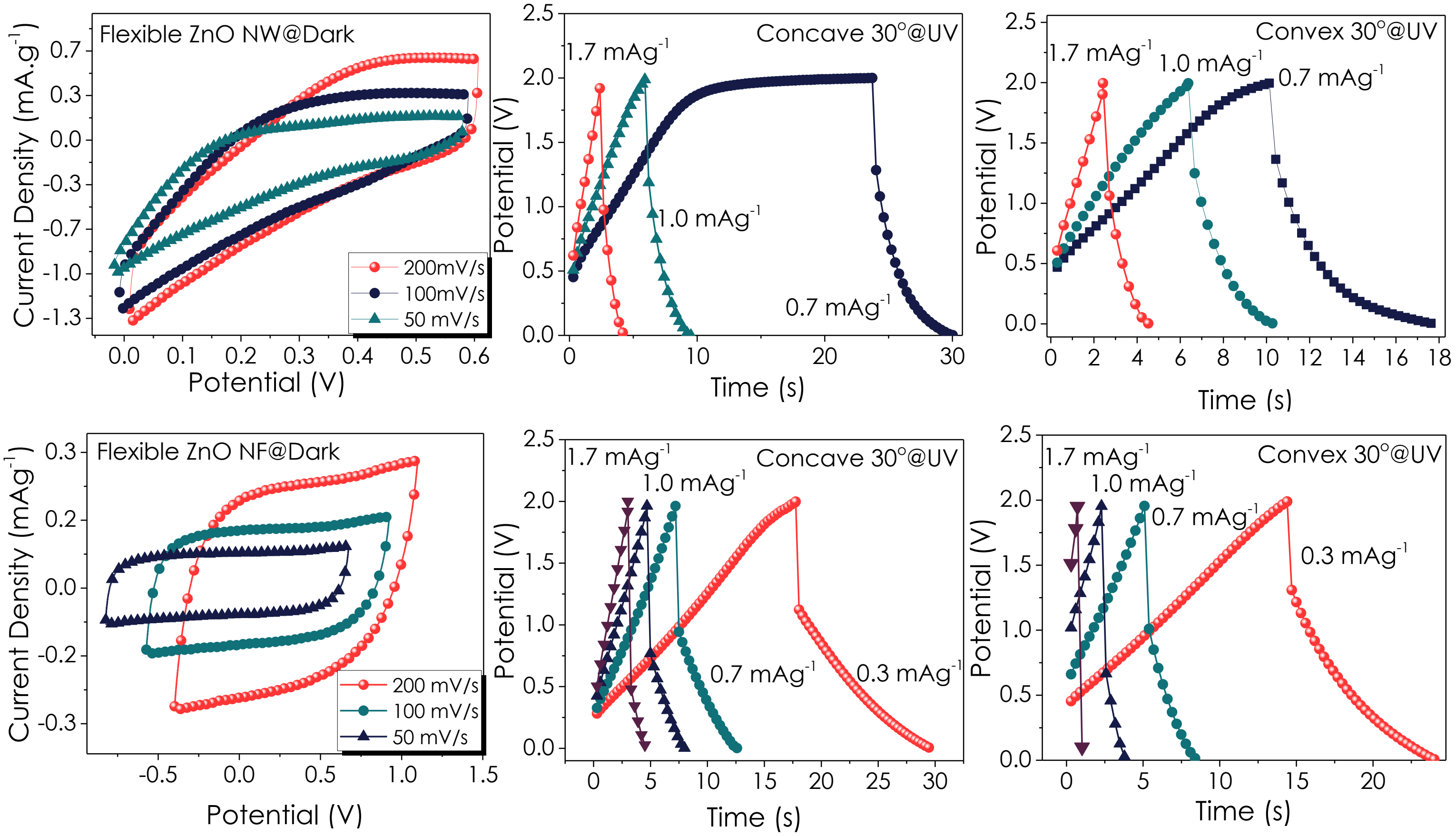

Figure S6. Cyclic voltammogram and galvanostatic charge-discharge curves of ZnO NW and NF

Comparison of energy and power density of rigid and flexible designs

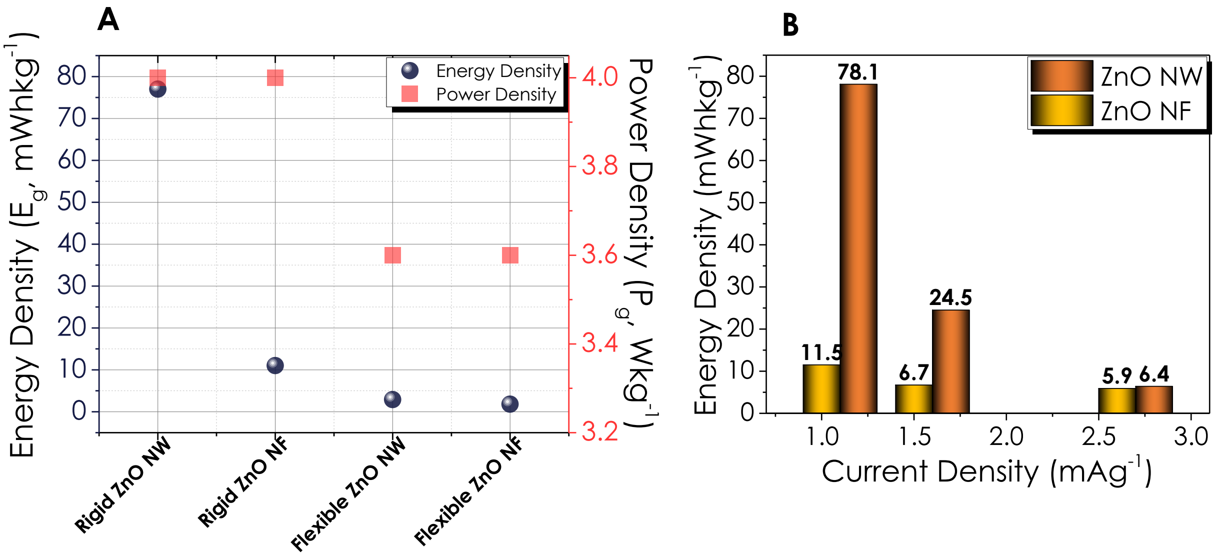

Figure S7. (A) Energy and power densities of ZnO-based rigid and flexible P-SCs, (B) Energy density versus current density plot for rigid P-SCs.

Electrochemical impedance spectroscopy-Bode graphs at Dark

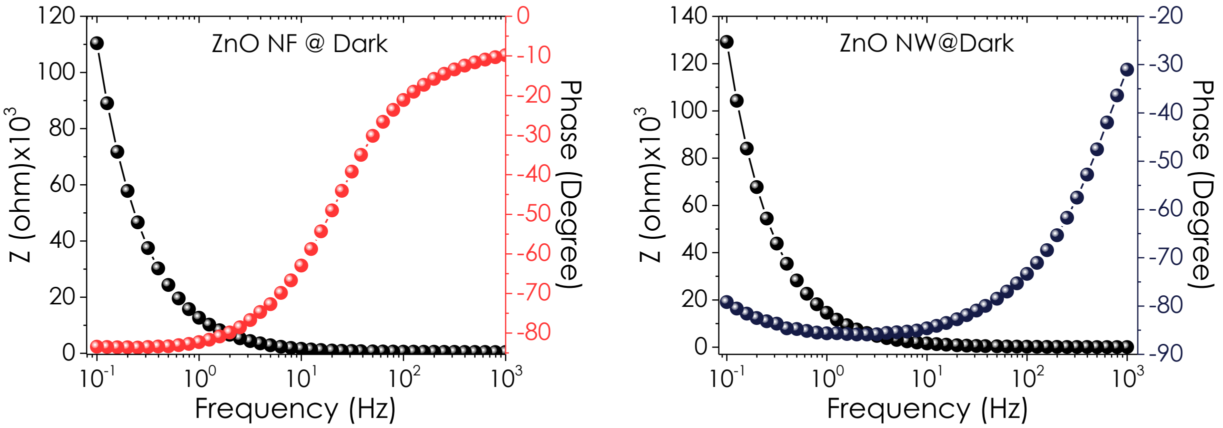

Figure S8. Bode curves at dark conditions

Frequency and bias voltage-dependent capacitance measurements

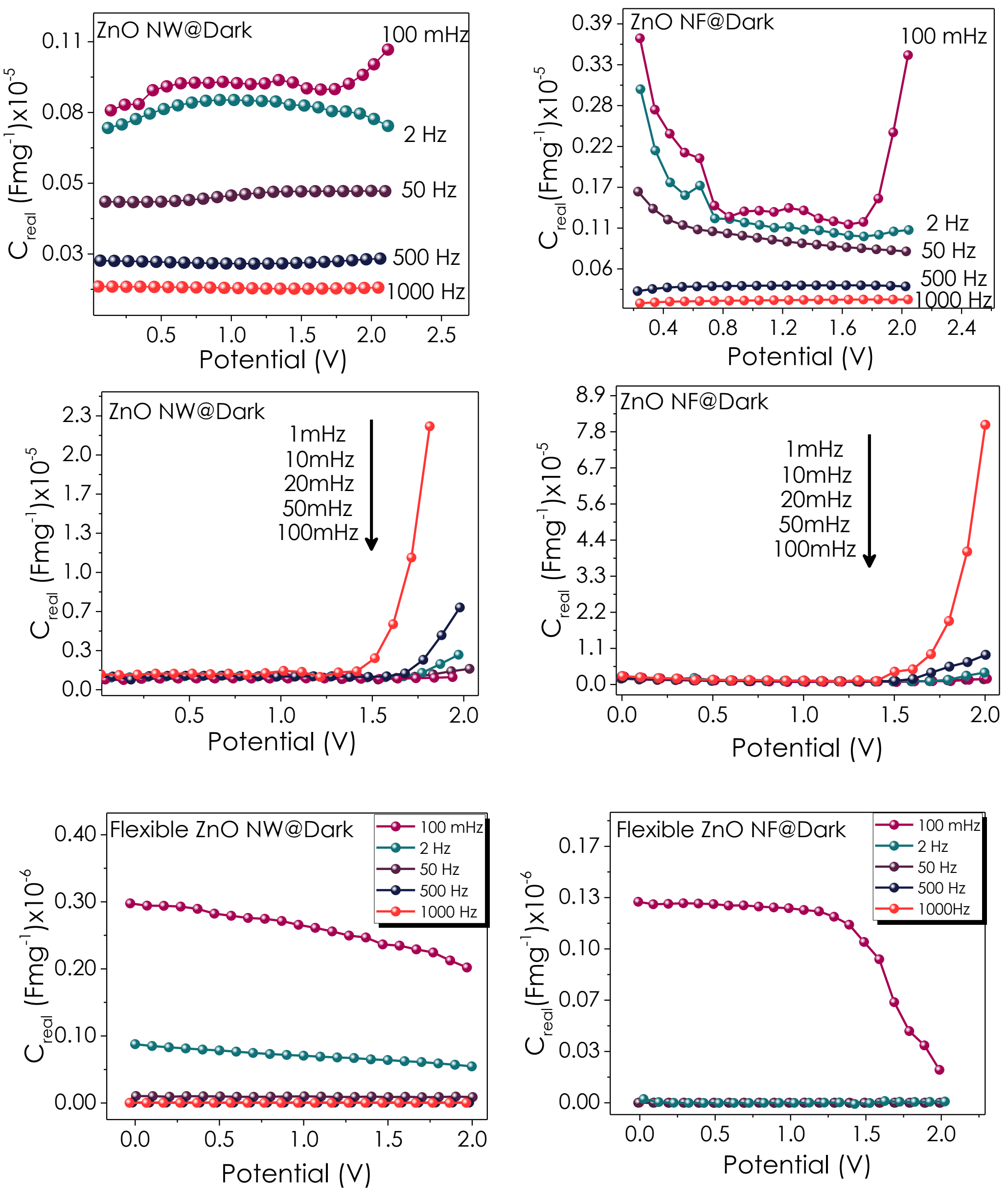

Figure S9. Frequency and applied bias voltage-dependent real capacitance plots ZnO NW and NF.

References

[1] X. Lu, M. Yu, T. Zhai, G. Wang, S. Xie, T. Liu, C. Liang, Y. Tong, Y. Li, *Nano Lett.* **2013**, *13*, 2628.

[2] H. Niu, D. Zhou, X. Yang, X. Li, Q. Wang, F. Qu, *J. Mater. Chem. A* **2015**, *3*, 18413.

[3] S. Selvam, J. H. Yim, *J. Mater. Chem. A* **2021**, *9*, 14319.

[4] N. Jayababu, D. Kim, *Nano Energy* **2021**, *82*, 105726.
